# ATP consumption in molecular reactions of neuronal signaling

**DOI:** 10.1186/1471-2202-15-S1-P179

**Published:** 2014-07-21

**Authors:** Nikon Rasumov, Erik De Schutter

**Affiliations:** 1 Okinawa Institute of Science and Technology, 1919-1 Tancha Onna-son, Okinawa, 904-0495 Japan

## 

The human brain consumes 10^6^ times less energy than the currently fastest supper computer [1], while maintaining a comparable performance in many demanding task [2]. These energetic efficiency has been suggested to result from primitive computations on a molecular level [3]. However, while the importance of ion channels on energy efficiency has been the primary focus of research [4,5], most computations occur at the molecular level prior to the amplification step and prior to the information transmission. We calculate the amount of energy consumed by such computations and compare their structural and functional properties. As a starting point, we chose the molecular reactions involved in long term depression and using our stochastic model [6] estimate the molecular energy consumption. To compare our feedback loop we investigate the energy consumption of millions of feedback loops in molecular signaling. For the first time we are able to go beyond the current size limit of 15 steps [7] and, using a computer cluster, detect feedback loops with hundreds of molecular reactions. We find that the number of ATPs consumed is related with size of positive feedback loop. We conclude that the energy consumed by the long term depression is only marginally above the physical limit of storing information and higher than its silicon equivalent of random access memory. Hence, this study provides the first systematic attempt to investigate the energy consumption of information-storing primitive computations and points towards energy efficient motifs for synthetic biology.

## References

[B1] Niven JE Laughlin SB Energy limitation as a selective pressure on the evolution of sensory systems Journal of Experimental Biology 2008 15 11 1792 1804 10.1242/jeb.017574 18490395

[B2] Ferrucci DA Introduction to "This is Watson" Ibm Journal of Research and Development 2012 15 3-4 15

[B3] Mead C Neuromorphic Electronic Systems Proceedings of the Ieee 1990 15 10 1629 1636 10.1109/5.58356

[B4] Sengupta B Stemmler M Laughlin SB Niven JE Action Potential Energy Efficiency Varies Among Neuron Types in Vertebrates and Invertebrates Plos Computational Biology 2010 15 7 10.1371/journal.pcbi.1000840 PMC2895638 20617202

[B5] Alle H Roth A Geiger JRP Energy-Efficient Action Potentials in Hippocampal Mossy Fibers Science 2009 15 5946 1405 1408 10.1126/science.1174331 19745156

[B6] Antunes G De Schutter E A Stochastic Signaling Network Mediates the Probabilistic Induction of Cerebellar Long-Term Depression J Neurosci 2012 15 27 10.1523/JNEUROSCI.5976-11.2012 PMC6622230 22764236

[B7] Ma'ayan A Cecchi GA Wagner J Rao AR Iyengar R Stolovitzky G Ordered cyclic motifs contribute to dynamic stability in biological and engineered networks Proc Natl Acad Sci U S A 2008 15 49 19235 19240 10.1073/pnas.0805344105 19033453 PMC2614745

